# Variation in heteroploid reproduction and gene flow across a polyploid complex: One size does not fit all

**DOI:** 10.1002/ece3.7791

**Published:** 2021-06-29

**Authors:** Brittany L. Sutherland, Laura F. Galloway

**Affiliations:** ^1^ Department of Biology University of Virginia Charlottesville VA USA; ^2^ Department of Biology University of Louisiana at Lafayette Lafayette LA USA

## Abstract

Whole‐genome duplication is considered an important speciation mechanism in plants. However, its effect on reproductive isolation between higher cytotypes is not well understood. We used backcrosses between different ploidy levels and surveys of mixed‐ploidy contact zones to determine how reproductive barriers differed with cytotype across a polyploid complex. We backcrossed F1 hybrids derived from 2X‐4X and 4X‐6X crosses in the *Campanula rotundifolia* autopolyploid complex, measured backcross fitness, and estimated backcross DNA cytotype. We then sampled four natural mixed‐ploidy contact zones (two 2X‐4X and two 4X‐6X), estimated ploidy, and genotyped individuals across each contact zone. Reproductive success and capacity for gene flow was markedly lower for 2X‐4X than 4X‐6X hybrids. In fact, 3X hybrids could not backcross; all 2X‐4X backcross progeny resulted from neotetraploid F1 hybrids. Further, no 3X individuals were found in 2X‐4X contact zones, and 2X and 4X individuals were genetically distinct. By contrast, backcrosses of 5X hybrids were relatively successful, particularly when crossed to 6X individuals. In 4X‐6X contact zones, 5X individuals and aneuploids were common and all cytotypes were largely genetically similar and spatially intermixed. Taken together, these results provide strong evidence that reproduction is low between 2X and 4X cytotypes, primarily occurring via unreduced gamete production, but that reproduction and gene flow are ongoing between 4X and 6X cytotypes. Further, it suggests whole‐genome duplication can result in speciation between diploids and polyploids, but is less likely to create reproductive barriers between different polyploid cytotypes, resulting in two fundamentally different potentials for speciation across polyploid complexes.

## Graphical abstract

To assess the role of ploidy in determining reproductive isolation and speciation in polyploid contact zones, we used backcrosses between different ploidy levels and surveys of mixed‐ploidy contact zones to determine how reproductive barriers differed with cytotype across a polyploid complex. Reproductive success and capacity for gene flow was markedly higher for 4X‐6X hybrids than 2X‐4X hybrids, which was also seen in natural mixed‐ploidy contact zones. Our results suggest whole‐genome duplication can result in speciation between diploids and polyploids, but is less likely to create reproductive barriers between different polyploid cytotypes, resulting in two fundamentally different potentials for speciation across polyploid complexes.

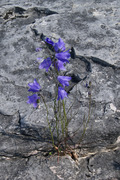

## 1 INTRODUCTION

Whole‐genome duplication has long been considered an important mechanism of plant speciation due to its ability to rapidly induce strong postzygotic isolation between newly emerged polyploids and their diploid progenitors (e.g., “triploid block”; Marks, 1966). The strength and consistency of this pattern has led to whole‐genome duplication being considered a means of “instantaneous” speciation, and it is cited as one of the clearest examples of sympatric speciation (Coyne & Orr, 2004). In part because of this strong reproductive isolation, whole‐genome duplication has historically been considered a critical driver of speciation with potential to influence patterns of diversification across angiosperms (Soltis et al., 2009).

Although reproductive isolation between diploids and tetraploids has been well studied and is well supported across numerous polyploid systems (Husband et al., 2016; Köhler et al., 2010; Kolár et al., 2009; Levin, 1978; Ramsey & Schemske, 1998), less attention has been paid to reproductive isolation among higher order polyploids. The few studies published to date suggest that reproductive isolation may be smaller between polyploid cytotypes than between diploids and tetraploids (Greiner & Oberprieler, 2012; Hülber et al., 2015; Sutherland & Galloway, 2017). These lower postzygotic barriers are manifest as hybrid formation between tetraploids and hexaploids in crossing experiments. However, hybridization between cytotypes, that is, heteroploid hybridization, is simply one barrier to genetic mixing. If heteroploid hybrids cannot then reproduce with parental cytotypes, these hybrids may merely serve as a reproductive sink and reduce gene exchange between the cytotypes. To fully understand whether higher order polyploids are less isolated than diploids and tetraploids requires evaluating the potential to produce viable and fertile backcrosses.

Furthermore, reproductive isolation is the product of a number of individual prezygotic and postzygotic reproductive barriers (Ramsey et al., 2003). As such, the ability of two cytotypes to create hybrids may not predict the probability that hybrids will be formed under natural conditions. Spatial structuring of cytotypes (Hülber et al., 2009; Husband & Schemske, 2000), pollinator preference between cytotypes (Kennedy et al., 2006; Thompson et al., 2004), and discrimination against heteroploid pollen relative to homoploid pollen (Koutecký et al., 2010) can create additional isolation. All else being equal, populations with more reproductive barriers will experience less gene exchange and will diverge more rapidly than those with fewer barriers. Postzygotic barriers between polyploid cytotypes can be smaller than those between diploids and polyploids (Sutherland & Galloway, 2017), and reproduction between polyploid cytotypes has been documented in some systems (Hülber et al., 2015; Sonnleitner et al., 2016; Laport et al., 2016). However, it is not known whether higher ploidy cytotypes experience more heteroploid gene exchange than diploids and tetraploids in natural populations and may therefore have lower rates of divergence and speciation.

The *Campanula rotundifolia* autopolyploid complex is a tractable system in which to investigate how whole‐genome duplication affects reproductive barriers and how such barriers shape gene flow across multiple cytotypes. This polyploid complex comprises three dominant cytotypes, with multiple contact zones between them, and greater reproductive isolation between diploids and tetraploids than between higher order polyploids (Sutherland & Galloway, 2017). We employ both heteroploid backcrosses and surveys of mixed‐ploidy contact zones to determine the extent to which barriers shape reproduction between cytotypes and whether patterns of heteroploid reproduction manifest as gene flow in natural contact zones. Specifically, we ask the following questions: (1) Are heteroploid hybrids capable of reproducing with parental cytotypes? (2) Does reproduction between cytotypes occur in *C. rotundifolia* mixed‐ploidy contact zones? and (3) are heteroploid reproduction and gene flow more common between tetraploids and hexaploids than between diploids and tetraploids?

## 2 METHODS

### 2.1 Study system

**FIGURE 1:**
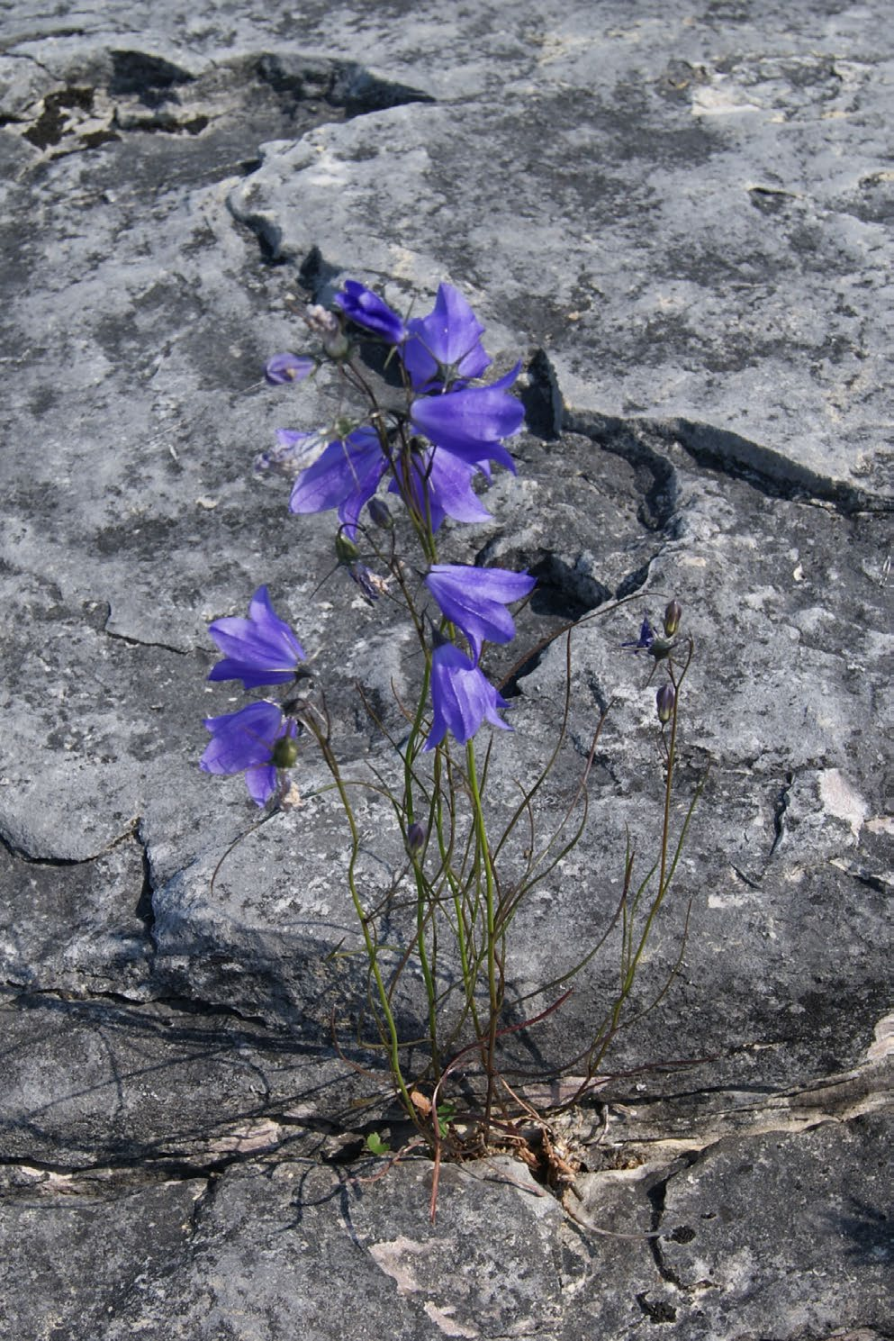
*Campanula rotundifolia* individual in alvar habitat on Manitoulin Island, Ontario, Canada

*Campanula rotundifolia* is a generalist pollinated perennial wildflower that favors calcareous soils (Figure 1). It has a broadly circumboreal distribution, located in the northern latitudes of North America and throughout much of Europe (Shetler, 1982; Stevens et al., 2012). It is an autopolyploid complex (Kovanda, 1966; Mansion et al., 2012) with three dominant cytotypes, diploid (2n = 34 chromosomes), tetraploid (2n = 68 chromosomes), and hexaploid (2n = 102 chromosomes; Kovanda, 1966; Stevens et al., 2012). Cytotypes are not uniformly distributed; tetraploids are common throughout the range, diploids are mostly restricted to northern and central Europe, and hexaploids are restricted to the western British Isles, central and western North America, and small populations in central Europe (Shetler, 1982; Stevens et al., 2012; Sutherland & Galloway, 2018; K. Šemberová, pers. comm.). Most populations have only one cytotype, though there are known diploid–tetraploid and tetraploid–hexaploid contact zones (Shepherd, 2007; Wilson et al., 2020; K. Šemberová, pers. comm.). Tetraploid and hexaploid populations have arisen via whole‐genome duplication multiple times in both Europe and North America (Sutherland & Galloway, 2018).

### 2.2 Backcrosses

To assess potential for gene flow between cytotypes, we conducted backcrosses between previously generated 2X‐4X and 4X‐6X F1 hybrids (Sutherland & Galloway, 2017) and their parental populations. Two sets of crosses were used to test each ploidy combination. Hybridized populations were chosen to be geographically close, to approximate plants sympatric in nature, except for one slightly more distant but genetically similar pair (Table S1). Heteroploid hybrids were created from reciprocal crosses. F1s showed parent‐of‐origin effects in germination but not postgermination traits, for example, pollen fertility and overall fitness (Sutherland & Galloway, 2017), so reciprocal F1 hybrids were pooled. 2X‐4X crosses produced triploid hybrids in sufficient numbers to be used in backcrossing. Additionally, these crosses produced tetraploid hybrids, indicating the likely contribution of a nonreduced gamete from the 2X parent. 4X‐6X crosses produced many fertile pentaploid hybrids (Sutherland & Galloway, 2017).

**FIGURE 2:**
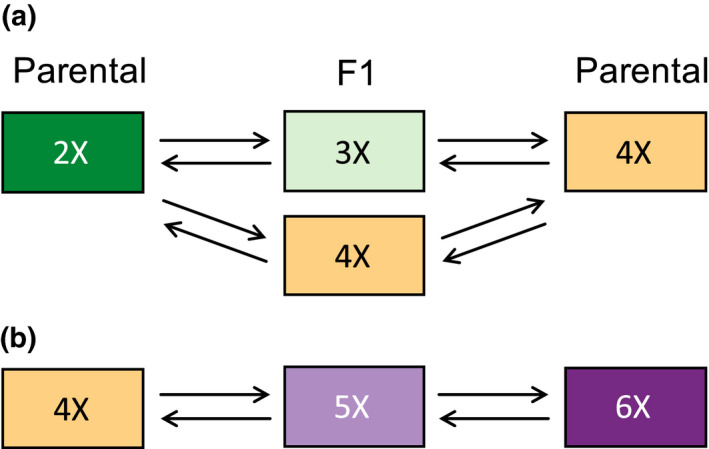
Schematic diagram of crossing design. F1 plants (middle column) were reciprocally crossed to both parental cytotypes. Two F1 cytotypes were crossed against parental 2X and 4X cytotypes (a), while only one F1 cytotype was crossed against 4X and 6X parental cytotypes (b). Crosses were repeated for both F1 hybrids per cytotype and for two replicates per arrow

Each F1 heteroploid hybrid was reciprocally backcrossed to two individuals from each parental population, resulting in eight crosses per F1 plant (Figure 2). Ten individuals were used for 4X and 5X hybrids, but only six for 3X hybrids due to poor germination. Two pollinations were conducted for each cross. This resulted in up to 160 planned pollinations per cytotype for 4X and 5X F1s (2 hybrid crosses × 10 F1 plants/cross × 2 directions × 2 parental populations × 2 pollinations), but only 96 planned pollinations for the 3X F1s. Due to insufficient flowers on some plants, actual pollination numbers were lower, but at least 90% of planned pollinations were performed (Table S2). Pollen fertility varied with cytotype; triploid hybrids produced approximately 34% viable pollen, tetraploids were fully pollen fertile, and pentaploids had approximately 75% pollen viability (Sutherland & Galloway, 2017). Hybrid plants with the highest pollen fertility were crossed, providing a maximal estimate of backcrossing potential for each population. To prohibit selfing prior to pollination, maternal flowers were emasculated by removing anthers in the bud (Sutherland & Galloway, 2018). During pollination, a surplus of pollen was brushed from a paternal flower onto the stigmatic lobes of a maternal flower. Mature fruits were collected just prior to dehiscence. Intrapopulation crosses were also conducted for each parental population at the same time as backcrosses.

Backcross success was measured using seed number and germination proportion. All fully developed brown seeds were counted. Then, up to ten seeds per fruit were planted in two replicates of five for both backcrosses and intrapopulation crosses. If a fruit produced <10 seeds, all seeds were sown. Only 22 seeds were obtained from the 88 backcrosses between diploids and triploids; these were sown individually. Seeds were germinated on a 12‐hr/12‐hr light/dark cycle at 22℃/15℃. Germination was scored every two days for six weeks, and then, germinants were randomly thinned to one per replicate and grown for an additional eight weeks to obtain sufficient leaf tissue for cytometric analysis.

### 2.3 Statistical analysis

Standardized seed set and germination were calculated to account for differences among populations. Backcross seed set was standardized by dividing the seed set of each fruit for each plant by the average seed set of intrapopulation crosses for the maternal population, and germination was standardized by dividing the mean germination rate of seeds for each replicate by the germination rate of seeds from intrapopulation crosses averaged across both parental populations. As previous work found high survival of all heteroploid F1 hybrids (Sutherland & Galloway, 2017), we focused on seed traits and calculated composite fitness as the product of relative seed set and relative germination.

We used a generalized mixed model to assess variation in fitness components. Fixed effects included F1 cytotype (3X, 4X, or 5X), parental cytotype (2X or 4X, 4X or 6X), backcross crossing direction (F1 hybrid as the maternal or paternal parent), and all possible interactions. Population (nested within F1 cytotype) was a random effect.

### 2.4 Mixed ploidy population sampling

To evaluate potential differences in gene flow between cytotypes in nature, four contact zones known to contain two ploidy levels were sampled and assessed for cytotype and genetic variation. Two 2X‐4X contact zones were located in central Europe: one in Mittelndorf in eastern Germany and one in Prague, Czech Republic (Table S1). These are likely contact zones, rather than in situ duplication events because chloroplast haplotypes differ between cytotypes (Sutherland & Galloway, 2018). The contact zones were small, discrete populations and were comprehensively surveyed, with GPS location and leaf tissue taken from all plants that were at least 1 m apart (38 and 30 plants, respectively).

Two 4X‐6X contact zones were sampled using transects due to their larger population sizes. For Cheddar Gorge in England (Table S1), plants were widespread over much of a 2‐km long limestone gorge, with cliffs up to 137 m high. Collection efforts consisted of four east–west transects: along both northern and southern rims, and along the northern and southern sides of the gorge bottom to a height of 2 m. In total, 122 individuals were sampled. In Misery Bay Provincial Park in Canada (Table S1), plants occur in patchy distributions on exposed limestone bedrock (alvar glades) in an otherwise heavily forested area. Plants were collected along an approximately 1‐km transect between the park entrance and Lake Huron that traversed two large glades. A total of 50 individuals were sampled. As with the 2X‐4X samples, Misery Bay in Canada is likely a contact zone because the chloroplast haplotype differs between cytotypes (Sutherland & Galloway, 2018). However, chloroplast haplotypes are the same for 4X and 6x individuals in Cheddar Gorge in England (Sutherland & Galloway, 2018), suggesting that the 6x individuals result from an in situ ploidy change or that cytotypes in the contact zone are less differentiated.

### 2.5 Flow cytometry and cytometric analysis

Flow cytometry was used to estimate ploidy level of both backcross progeny and wild‐collected plants from each contact zone. For backcross progeny, approximately 30 mg of fresh tissue was analyzed from a total of 240 plants, 15 from each cross type, representing 40% of backcrosses from 2X‐4X F1s and 19% of those from 4X‐6X F1s. For wild‐collected plants, approximately 10 mg of silica‐dried tissue was used for analysis. Flow cytometric analysis followed a modified Otto 2‐step protocol (Otto, 1990; see Sutherland & Galloway, 2017, for details). Prior to visualization, samples were treated with 50 ng/μl propidium iodide (PI) and 50 ng/μl RNase I and then processed using a BD FACSCalibur Cell Analyzer equipped with a 488 nm laser. Relative PI fluorescence at maximum peak height was compared to external standards; radish (*Raphanus sativus “Saxa”: DNA content 1.11 pg/2C)* was used for backcrosses containing diploid parents and for 2X‐4X contact zones, and soybean (*Glycine max “Polanka”: DNA content 2.50 pg/2C)* for backcrosses containing hexaploid parents and for 4X‐6X contact zones.

DNA content was estimated by comparing the relative fluorescence of unknown samples to that of external standards. The external standards were run at the start of every analytical session and re‐run following any changes in calibration. To assign a cytotype to each individual, estimated DNA content for each plant was compared to a known diploid *C. rotundifolia* population (population 23; Table S1). For all backcross progeny and all mixed‐ploidy populations except Cheddar Gorge, discrete gaps in the distribution of DNA content ratios were used to bin individuals into euploid or aneuploid categories, as has been employed in other studies (Čertner et al., 2017; Sonnleitner et al., 2010). Because DNA content ratios did not bin discretely in Cheddar Gorge (see also Wilson et al., 2020), means and standard deviations of euploid tetraploid and hexaploid populations were calculated, and a cutoff of 3 standard deviations from the mean was used to assign individuals as euploid tetraploids or hexaploids. These align with DNA content ranges previously reported for *C. rotundifolia* (Wilson et al., 2020). Any values outside these cutoffs were assigned as putative aneuploids and pentaploids.

### 2.6 DNA extraction, microsatellite amplification, and analysis

Microsatellite loci were used to determine genetic similarity between individuals and cytotypes within each mixed‐ploidy contact zone. DNA was extracted from all samples using a CTAB protocol optimized for plate processing (Costa & Roberts, 2014). Eight microsatellite markers specifically designed for *C. rotundifolia* (Plue et al., 2015; Table S3) were chosen for amplification and analysis. Microsatellite loci were amplified as duplexes using 5′‐fluorescently labeled M13 adapters annealed to forward primers. Amplified loci were visualized at the Yale Genome Sequencing Center and scored using GeneMarker 3.4 software.

The genetic similarity of cytotypes within each contact zone was determined. First, alleles private to a given cytotype in a given population were counted and then standardized for the total population size using rarefaction analysis via HP‐RARE 1.0 (Kalinowski, 2005). Then, an investigation of genetic distance was performed using POLYSAT 1.7 (Clark & Jasieniuk, 2011). Pairwise genetic distances were first calculated for all individuals within a contact zone using the Bruvo distance function (which accounts for allelic mutations), and then, a principal coordinates analysis (PCoA) was performed on these pairwise distances.

## 3 RESULTS

### 3.1 Backcross fitness

F1 backcross direction (whether the F1 served as maternal or paternal parent) did not affect seed set, germination, or composite fitness for any crosses (Table 1). Therefore, all data reported are pooled across both backcross directions.

**TABLE 1:** Analysis of variance for backcrosses, testing the effects of F1 cytotype (3X, 4X, or 5X), parental cytotype (2X or 4X for 3X, 4X F1s; 4X or 6X for 5X F1s), and backcross direction (F1 plant as the mother or father) on seed set, germination, and composite fitness

Source	*df*	Seed set	Germination	Fitness
F1 Cytotype	3	16.76***	10.83***	15.81***
Parental Cytotype	2	16.49***	13.18***	19.03***
F1 × Parent	2	19.79***	9.80***	14.73***
Backcross Direction	1	0.47	1.37	1.22
F1 × Direction	3	0.49	1.82	0.83
Parent × Direction	2	0.45	3.85	0.97
Three‐Way Interaction	2	0.06	4.26	1.02
Error	145			

Note

Population, nested in F1 cytotype was included as a random effect (results not shown). *F*‐values listed.

*** *p*‐value < .0001, all others *p* > .05.

**FIGURE 3:**
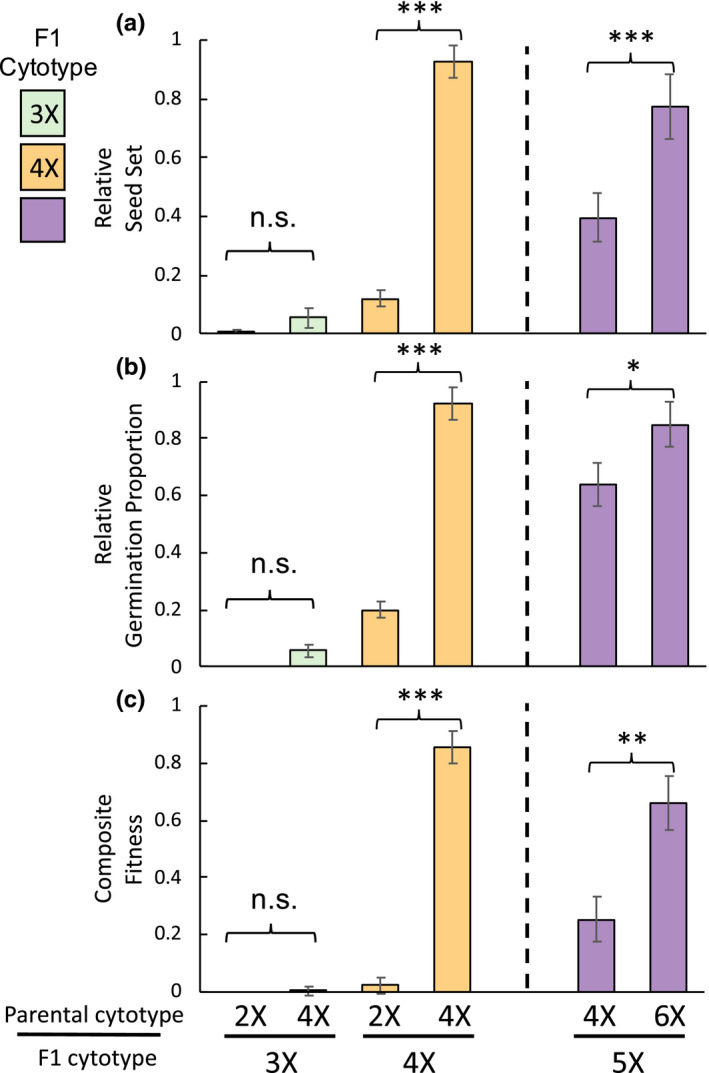
Performance of backcrosses of F1 hybrids between 2X‐4X or 4X‐6X and their parental populations: relative seed set (a), relative germination proportion (b), and composite fitness (c). Seed set standardized to the maternal population; germination standardized to both parental populations. The top row denotes the backcross parent cytotype, and the bottom row denotes the F1 hybrid parent cytotype. Colors differentiate F1 cytotypes; error bars denote standard error. Differences between backcrosses of the parental cytotypes as determined by orthogonal contrasts: ***p‐value < .0001, all others *p* > .05

Tetraploid hybrids from 2X‐4X crosses were highly successful when backcrossed to a tetraploid individual, but experienced poor offspring fitness when crossed with a 2X individual (F1 × Parent; Table 1). Backcrosses between tetraploid F1 hybrids and tetraploid individuals set approximately 7.7 times as many seed (Figure 3a) and germinated 4.6 times better than backcrosses to diploid individuals (Figure 3b). Overall, backcrosses between 4X F1 hybrids and 4X individuals were 86% as fit as intrapopulation crosses based on our composite fitness measure (Figure 3c). Tetraploid F1 backcrosses to diploid individuals, by contrast, had composite fitness that was near zero (Figure 3c).

Backcrosses involving triploids yielded no offspring that survived to adulthood. Backcrosses between triploid F1s and diploid individuals only produced 22 seeds (1% relative seed set), while backcrosses to tetraploid individuals produced 103 seeds total (5.5% relative seed set; Figure 3a). Backcrosses between triploid F1 and diploid individuals produced no germinants, and those to tetraploid individuals had only 6 germinants (5.8% germination rate; Figure 3b), none of which survived long enough to obtain tissue for flow cytometry.

Backcrosses involving pentaploid hybrids were more successful when crossed with hexaploids than with tetraploids (F1 × Parent; Table 1). When pentaploids were backcrossed to hexaploids, seed set was roughly double that of backcrosses to tetraploids (Figure 3a). Pentaploid F1 hybrids had 33% higher germination when backcrossed to hexaploids than to tetraploids (Figure 3b). Finally, pentaploids had approximately 2.5 times higher composite fitness when backcrossed to hexaploids than to tetraploids (Figure 3c).

### 3.2 Backcross cytotypes

**FIGURE 4:**
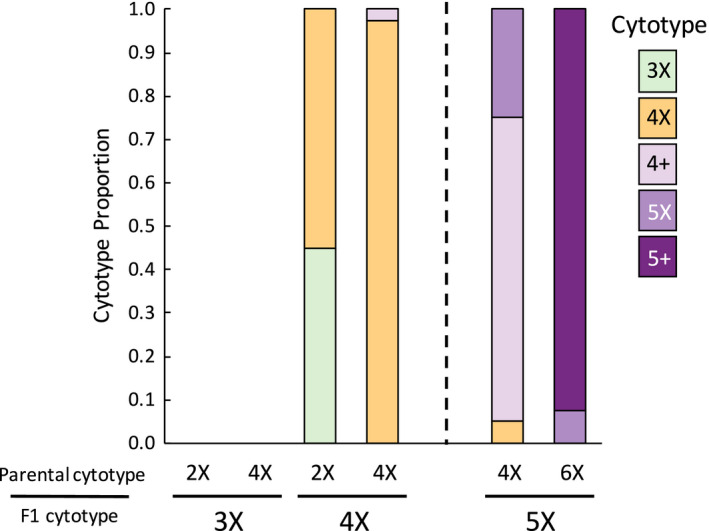
Cytotypic composition of backcross progeny. “+” denotes putatively aneuploid cytotypes. Cytotypes crossed shown on *x*‐axis

Coefficients of variation for backcross cytotypes averaged 4.37 ± 0.45%. The cytotypes of the progeny were more variable in backcrosses to the lower ploidy parent. Backcrosses between tetraploid F1s and tetraploid individuals produced almost exclusively tetraploid offspring, whereas backcrosses to diploids produced mixtures of triploid and tetraploid individuals (Figure 4). Progeny from 5X‐4X crosses showed considerable variation; 5% were consistent with tetraploids, 70% with aneuploids between 4X and 5X, and 25% with pentaploids (Figure 4). Progeny from 5X‐6X crosses were almost all aneuploid between pentaploid and hexaploid.

### 3.3 Contact zone cytotypes

**FIGURE 5:**
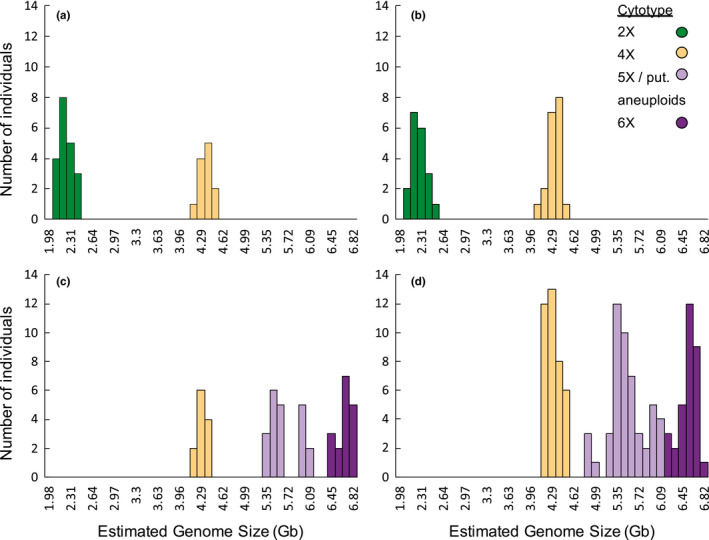
Cytotypic distribution of *Campanula rotundifolia* individuals in each contact zone. *X*‐axis denotes the estimated genome size. Colors denote assigned ploidy level based on genome size (see Methods for details; aneuploids between 4X and 6X were grouped with 5X). Contact zones are as follows: (a) Mittelndorf, (b) Prague, (c) Misery Bay, and (d) Cheddar Gorge

**FIGURE 6:**
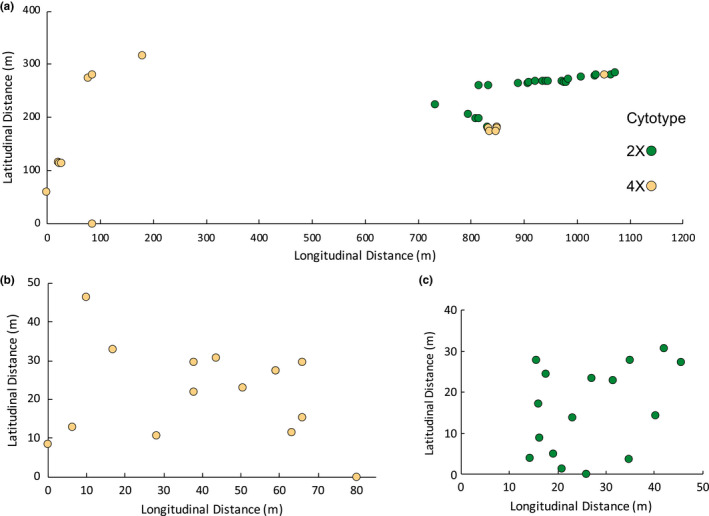
Spatial distribution of *C. rotundifolia* individuals in 2X‐4X contact zones in Mittelndorf, Germany, and Prague, Czechia. Due to the distance between diploids and tetraploids in Prague, Czechia, individuals are displayed in separate groups. The *x*‐axis denotes relative distance in meters along a latitudinal line, and the *y*‐axis denotes relative distance in meters along a longitudinal line

Coefficients of variation for backcross cytotypes averaged 5.18 ± 0.91%. Diploid–tetraploid contact zones comprised individuals that clustered discretely around a fluorescence intensity indicative of either diploidy or tetraploidy (Table 2; Figure 5a,b). In Mittelndorf, diploid individuals were primarily located along a path in a mown field in the northeast of the population while most tetraploid individuals were located peripherally to the south and west, although four were intermixed with diploids (Figure 6a). The Prague contact zone comprised two distinct subpopulations located approximately 8 km apart, each comprising only one observed cytotype (Figure 6b,c).

**TABLE 2:** Summary of population sampling and cytotype distribution in each of the four *Campanula rotundifolia* contact zones

Contact zone	Ploidy	Individuals	2X	4X	5X	6X
Mittelndorf	2X−4X	38	23	15		
Prague	2X−4X	30	16	14		
Cheddar Gorge	4X−6X	122		46	44	32
Misery Bay	4X−6X	50		12	21	17

Note

Aneuploids (individuals with estimated DNA content between 4X and 6X, see Methods) were included with the 5X ploidy class.

**FIGURE 7:**
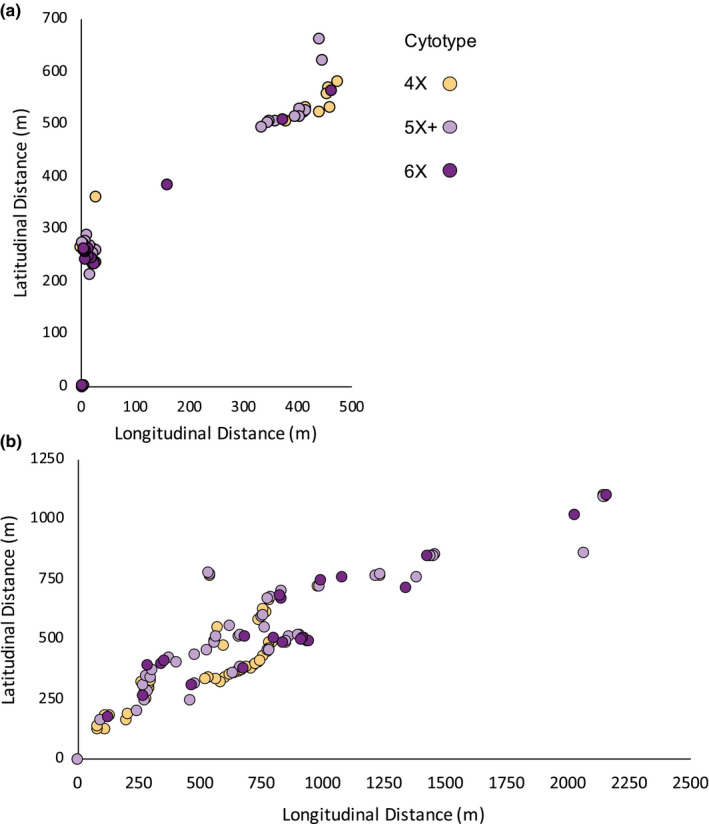
Spatial distribution of *C. rotundifolia* individuals in 4X‐6X contact zones in Cheddar Gorge, England, and Misery Bay Provincial Nature Reserve, Ontario, Canada. The *x*‐axis denotes relative distance in meters along a latitudinal line, and the *y*‐axis denotes relative distance in meters along a longitudinal line

In contrast, the two tetraploid–hexaploid contact zones contained numerous individuals of intermediate cytotype (Table 2; Figure 5c,d). 38% of all individuals in Cheddar Gorge were either pentaploid or aneuploid, primarily aneuploid between 5X and 6X. Ploidy levels at the base of the gorge were largely mixed, with no clustering of tetraploids or hexaploids (Figure 7b). However, individuals on the southern rim were almost exclusively tetraploid. Likewise, 42% of all individuals in Misery Bay were pentaploid or aneuploid, again with aneuploids more common between 5X and 6X. Tetraploids were primarily found in the northeastern glade while hexaploids were found to the southwest. Pentaploids and aneuploids were common in both glades (Figure 7a).

### 3.4 Genetic differentiation between cytotypes

3–18 alleles were recovered for each of the eight microsatellite loci amplified for each contact zone (Table 3). More alleles were recovered in 4X‐6X contact zones than 2X‐4X contact zones, but this may have been due to their larger size. However, the 2X‐4X contact zones had considerably more alleles that were private to a cytotype (Table 3). Within 2X‐4X contact zones, 4–6 private alleles were found within diploids and 3–9 within tetraploids (Table 3). By contrast, one private allele was found among tetraploids in either 4X‐6X contact zone, with zero or one private alleles found in the remaining cytotypic classes (Table 3).

**TABLE 3:** Summary statistics describing the allelic diversity in and genetic variation of microsatellite loci amplified for *Campanula rotundifolia's* four contact zones

Contact zone	Ploidy	Total alleles	Alleles/Locus	PA	PA_R_	1st Axis	2nd Axis
Mittelndorf	2X−4X	38	3.1	9	3.67	34.16%	8.47%
Prague	2X−4X	41	3.8	13	4.52	36.88%	11.91%
Cheddar Gorge	4X−6X	69	8	3	2.42	19.60%	13.07%
Misery Bay	4X−6X	60	7.1	1	1.35	11.77%	8.48%

Note

Ploidy refers to the dominant cytotypes present in the contact zone. PA is the count of alleles that are private to either cytotype in the contact zone; PA_R_ is the rarefied count of private alleles to standardize by population size. The percentage variance accounted for by the first two axes of principal coordinates analysis on the genetic distances between individuals in each contact zone is given.

**FIGURE 8:**
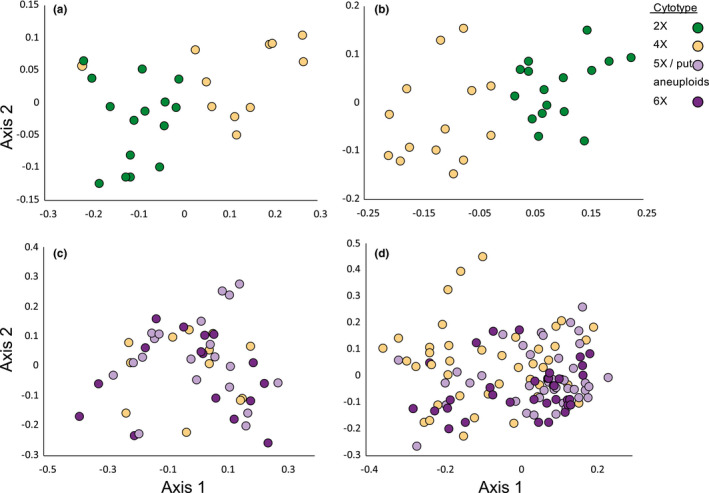
First two axes from a principal coordinates analysis of pairwise genetic distances between individuals in each of the *C. rotundifolia* four ploidy contact zones. (a) Mittelndorf and (b) Prague are 2X‐4X contact zones, and (c) Misery Bay and (d) Cheddar Gorge are 4X‐6X contact zones. Aneuploids between 4X and 6X were grouped with 5X

PCoAs showed differences in cytotypic clustering patterns between 2X‐4X and 4X‐6X contact zones. For both 2X‐4X contact zones, diploids and tetraploids formed separate clusters (Figure 8a,b), indicating that the cytotypes are genetically distinct and individuals within cytotypes are more similar to each other than to the other cytotype. By contrast, little clustering by cytotype was observed in either 4X‐6X contact zone (Figure 8c,d), indicating that cytotypes were not genetically distinct. Stronger clustering in the 2X‐4X contact zones is also seen in the first axis of the PCoA, which accounts for almost twice the variance in 2X‐4X contact zones as in 4X‐6X contact zones (Table 3).

## 4 DISCUSSION

The assumption that changes in ploidy are de facto speciation events has been long‐standing (e.g., Coyne & Orr, 2004), but the extent to which divergence and speciation are consistent across ploidy levels within a polyploid complex is less clear. To investigate divergence and potential for speciation within a polyploid complex, we evaluated reproductive isolation and gene flow in *Campanula rotundifolia* contact zones. Reproductive isolation was measured as a function of backcrossing ability of heteroploid hybrids, and gene flow inferred via genetic similarity of cytotypes and presence of intermediate cytotypes. We found that whole‐genome duplication from diploidy to tetraploidy in *Campanula rotundifolia* is largely consistent with the “instantaneous” speciation hypothesis. However, subsequent genome duplication events to higher cytotypes do not necessarily exhibit comparable heteroploid reproductive isolation. This pattern suggests that whole‐genome duplication does not have consistent effects on reproductive isolation across cytotypes within a polyploid complex; rather, the capacity for ploidy change to foster speciation may diminish as ploidy level increases.

Diploid–tetraploid gene flow via triploid intermediates is effectively nonexistent in *C. rotundifolia* due to limited hybrid formation (Sutherland & Galloway, 2017) and poor success of backcrosses of F1 hybrids to diploids and tetraploids. Previous work on diploid–tetraploid reproduction elucidated two common barriers: low viability of triploid hybrids (i.e., “triploid block”; Husband & Sabara, 2004; Marks, 1966) due to parental genomic imbalance (Köhler et al., 2010; Stoute et al., 2012) and low fertility of the hybrids due to unbalanced chromosomes during meiosis (Henry et al., 2005). Following these expectations, triploid *C. rotundifolia* exhibited poor fertility, though that conclusion is tempered somewhat by the limited number of triploids available. However, the extremely low backcross germination and complete lack of survival suggests additional barriers. Among other possibilities, likely sources of poor success include parental genomic imbalance in backcrosses to triploids, and aneuploid gametes from the triploid parents that experience fatal gene dosage irregularities (Birchler & Johnson, 2017; Henry et al., 2010).

A lack of triploids in natural population samples further supports the presence of total or near‐total reproductive isolation between diploids and tetraploids. The small size of sampled diploid–tetraploid populations leaves open the possibility that triploids form infrequently and were not sampled. However, the narrow distribution of estimated genome sizes for both diploids and tetraploids in 2X‐4X contact zones (indicating only euploid individuals), combined with the distinct genotypic clusters formed by diploids and tetraploids in both contact zones, indicates that any triploids formed are inviable or infertile and do not contribute to diploid–tetraploid gene flow.

In addition to the expected triploids, neotetraploids are also formed from diploid–tetraploid crosses. Despite serving as a conduit for heteroploid gene flow in other systems (Bringhurst & Gill, 1970; Kreiner et al., 2017; Ramsey, 2006), and despite neotetraploids having few fertility deficits, we do not find evidence that they contribute to gene flow. An apparent neotetraploid individual was found in the Mittelndorf contact zone (Figure 6b), but there was no genetic overlap between diploids and tetraploids in either mixed‐ploidy population, indicating that neotetraploids are not a conduit of gene flow.

In contrast, hybrids formed between tetraploids and hexaploids are fertile and contribute to gene flow between the cytotypes. Although pentaploid hybrids were not as fertile as parental cytotypes, viable backcross progeny were common, particularly backcrosses to hexaploids. Most of the backcross offspring of pentaploids are likely aneuploid, falling outside the typical euploid range of tetraploids, pentaploids, or hexaploids. Because aneuploids often exhibit reduced viability and fertility (Dujardin & Hanna, 1988; Ramsey & Schemske, 2002), it might be expected that these backcross offspring do not contribute to heteroploid gene flow. However, the wide range of genome sizes and lack of genotypic divergence between cytotypes in mixed tetraploid–hexaploid populations suggest that backcross offspring derived from pentaploids—especially in crosses to hexaploids—are fertile despite apparent aneuploidy. Pentaploids and aneuploids have also been reported in other *C. rotundifolia* 4x‐6x contact zones in England as well as in the Cheddar Gorge population (Wilson et al., 2020). Their repeated occurrence supports hybridization and backcrossing as their source, rather than a loss or gain of chromosomes during normal meiotic/mitotic processes in higher ploidy individuals (cf. Costich et al., 2010; Ramsey & Schemske, 2002).

The marked differences in backcross fitness and genetic divergence in mixed‐ploidy contact zones demonstrate that tetraploid and hexaploid *C. rotundifolia* are more likely to interbreed and share genes than diploids and tetraploids. One possible explanation is that tetraploid and hexaploid cytotypes in the contact zones are more genetically similar and therefore more compatible than the cytotypes in diploid–tetraploid contact zones. This is unlikely as the cytotypes are genetically similar for one diploid–tetraploid and one tetraploid–hexaploid contact zone and less so for the other contact zone (Sutherland & Galloway, 2018). Similar patterns of gene exchange between cytotypes, regardless of genetic divergence, suggest that differences in ploidy of the contact zones, rather than genetic distance, underlie the distinct barriers to hybridization. Furthermore, support for gene flow is found where intermediate cytotypes are present and not otherwise, supporting heteroploid gene exchange as the mechanism of genetic similarity. Studies in other taxa would indicate whether gene flow differences between diploids and polyploids versus between polyploid cytotypes is a general result.

The mechanisms behind ploidy‐mediated differences in reproductive barriers and the apparent asymmetrical gene flow observed here are poorly understood, but patterns of developmental irregularity in other systems may provide insight. Parentally imprinted small RNAs regulate development of endosperm. In diploid–tetraploid crosses, imbalance of these RNAs results in aberrant seed development and poor viability, and deficits tend to be less severe when the maternal parents has the higher ploidy (Haig & Westoby, 1988; Scott et al., 1998; Stoute et al., 2012). In the tetraploids and hexaploids, the magnitude of this imbalance is typically smaller (Bauer, 2006), ameliorating the negative effects on endosperm development and hybrid viability. A similar process may explain why pentaploids backcross more easily to hexaploids than tetraploids (see also Wilson et al., 2020). In crosses between a pentaploid or near‐pentaploid and a hexaploid, the magnitude of genomic imbalance in the developing hybrid endosperm is expected to be less than that between a pentaploid and a tetraploid. Although this mechanism has not been confirmed, similar patterns of asymmetric gene flow toward the larger cytotype have been found in *Senecio carniolicus* (Hülber et al., 2015; Sonnleitner et al., 2010).

The contrasting patterns of reproductive isolation and heteroploid gene flow found in the *C. rotundifolia* complex may help explain the cytotype spatial distributions in mixed‐ploidy contact zones. Diploid and tetraploids are not only genetically distinct, but spatially separated into mostly single‐ploidy clumps in contact zones. In contrast, tetraploids and hexaploids were generally spatially and genetically intermixed. The spatial separation of diploids and tetraploids in contact zones, relative to the more intermixed tetraploid–hexaploid contact zones, reduces the probability of gene exchange. Other intermixed tetraploid–hexaploid zones have been reported in *C. rotundifolia* (McAllister, 1972; Wilson et al., 2020), but similarly interspersed diploid–tetraploid contact zones are not known to exist. Spatial clumping of diploids and tetraploids may also reflect local adaptation to different microhabitats, although the lack of any such clumping from similarly divergent tetraploids and hexaploids as in Misery Bay suggests that diploid–tetraploid and tetraploid–hexaploid contact zones exhibit different patterns of gene flow. Although the numbers are not large, this pattern suggests that diploid–tetraploid incompatibility may, via reinforcement (Ortiz‐Barrientos et al., 2009) or minority cytotype exclusion (Husband et al., 2000), result in spatial separation between cytotypes. In contrast, the compatibility among polyploids may create conditions permissive for truly sympatric contact zones that facilitate further genetic mixing. These disparate spatial patterns may further exacerbate differences in gene flow, or encourage different patterns of local adaptation that may spur divergence between diploids and tetraploids but constrain it between higher cytotypes.

Numerous intrinsic and extrinsic factors contribute to gene flow and, in turn, to divergence and speciation. However, in *C. rotundifolia*, extrinsic factors appear to exacerbate the difference in intrinsic reproductive barriers observed between diploids and polyploids compared to higher polyploids. Reproductive barriers between parental populations, as well as between those populations and heteroploid hybrids, differed with cytotype, with parental and F1 barriers stronger between diploids and tetraploids than between different polyploids. Pollinators show a preference for rare cytotypes in this system, which increases the likelihood of gene flow between a rare cytotype and a common one in contact zones (Sutherland et al., 2020). This preference, coupled with differences in reproductive barriers across cytotypes, sets up two expectations for *C. rotundifolia* contact zones. In diploid–tetraploid contact zones, the increased potential for heteroploid gene exchange due to a pollinator preference for rarity is blunted by the strong intrinsic reproductive barriers between diploids and tetraploids. In contrast, the more permissive reproductive barriers between tetraploids and hexaploids, coupled with pollinator preference for rarity, further drives heteroploid gene exchange. Although pollinator behavior, spatial structure, and demography may be idiosyncratic to system, polyploid complexes with similar ecologies and intrinsic barriers may likewise show increased gene exchange between different polyploid cytotypes than between diploids and polyploids.

Although polyploids have been known to exist in taxonomically related, potentially interbreeding complexes for a century (Blakeslee et al., 1920), the effect of polyploidy on speciation and angiosperm diversification remains the subject of active debate (Soltis et al., 2009; Mayrose et al., 2011; Wood et al., 2009; Arrigo & Barker, 2012). The differences in heteroploid reproduction and gene flow observed here provide one explanation for the lack of clarity on the contribution of polyploidy to species richness. Gene flow homogenizes populations and can constrain speciation by slowing the accumulation of genetic incompatibilities (Fitzpatrick et al., 2009). However, due to long‐standing assumptions of strong heteroploid reproductive barriers across cytotypes, heteroploid gene flow has largely been discounted. This study demonstrates that heteroploid gene flow within a polyploid complex can be frequent between higher cytotypes but rare or nonexistent between diploids and tetraploids, though limited natural population sizes curtailed sampling. If such a pattern is common across angiosperms, it suggests that at least two rates of speciation may be present within polyploid complexes: a higher rate between diploids and tetraploids, and a lower rate between cytotypes higher than diploids. Such variation in heteroploid gene flow may help explain why polyploidy appears to be an active driver of speciation and diversification while manifesting in numerous hybrid and cytotype complexes.

## CONFLICT OF INTEREST

None declared.

## AUTHOR CONTRIBUTIONS

**Brittany L. Sutherland:** Conceptualization (lead); data curation (lead); formal analysis (equal); investigation (equal); methodology (equal); writing‐original draft (lead); writing‐review & editing (equal). **Laura F. Galloway:** Conceptualization (supporting); data curation (supporting); formal analysis (equal); funding acquisition (lead); methodology (equal); project administration (lead); supervision (lead); writing‐original draft (supporting); writing‐review & editing (equal).

## Supporting information

Table S1‐S3

## Data Availability

Data from this study have been deposited in Dryad (https://doi.org/10.5061/dryad.gqnk98smp).
