# Supplementary material for: Variation in heteroploid reproduction and gene flow across a polyploid complex: One size does not fit all
Source: Ecol Evol. 2021 Jun 29;11(14):9676–88. doi: 10.1002/ece3.7791 (PMC8293777; doi:10.1002/ece3.7791)
Supplement: Supplementary file 1 — Table S1‐S3 [file ECE3-11-9676-s001.docx]

Table S1: *Campanula rotundifolia* populations used for (A) backcrossing and (B) mixed-ploidy contact zone surveys. Each pair used in backcrosses is delineated by a horizontal line.

Table S2: F1 interploid hybrids used in backcrosses. Number of backcrosses performed. See Table S1 for population locations.

Table S3) Microsatellite loci used for gene flow assessment. All loci taken from Plue et al., 2015.

| Microsatellite Loci |
| --- |
| Camrot_002896 |
| Camrot_003772 |
| Camrot_010189 |
| Camrot_010246 |
| Camrot_011624 |
| Camrot_013423 |
| Camrot_015251 |
| Camrot_019708 |
